# Hypermutability of *Mycolicibacterium smegmatis* due to ribonucleotide reductase-mediated oxidative homeostasis and imbalanced dNTP pools

**DOI:** 10.1080/22221751.2025.2480698

**Published:** 2025-03-18

**Authors:** Xiao Zhang, Yuchang Di, Yu Zhang, Youwei Hu, Mingzhe Chi, Jian Kang, Yuqing Zheng, Hengyu Wang, Yu Wang, Jiazhen Chen, Xuelian Zhang

**Affiliations:** a State Key Laboratory of Genetic Engineering, School of Life Sciences, Fudan University, Shanghai, People’s Republic of China; b Shanghai Sci-Tech Inno Center for Infection & Immunity, Shanghai, People’s Republic of China; c Department of Infectious Diseases, Shanghai Key Laboratory of Infectious Diseases and Biosafety Emergency Response, National Medical Center for Infectious Diseases, Huashan Hospital, Shanghai Medical College, Fudan University, Shanghai, People’s Republic of China; d MOE Engineering Research Center of Gene Technology and Shanghai Engineering Research Center of Industrial Microorganism, Fudan University, Shanghai, People's Republic of China

**Keywords:** Mycobacterium, ribonucleotide reductase, oxidative homeostasis, dNTPs balance, genomic stability, mutation, drug resistance

## Abstract

Ribonucleotide reductase (RNR) catalyzes the synthesis of four deoxyribonucleoside triphosphates (dNTPs), which are essential for DNA replication. Although dNTP imbalances reduce replication fidelity and elevate mutation rates, the impact of RNR dysfunction on Mycobacterium tuberculosis (Mtb) physiology and drug resistance remains unknown. Here, we constructed inducible knockdown strains for the RNR R1 subunit NrdE in Mtb and Mycolicibacterium smegmatis (Msm). NrdE knockdown significantly impaired growth and metabolic imbalances, indirectly disrupting oxidative homeostasis and mycolic acid synthesis, while increasing levels of intracellular ROS accumulation and enhancing cell wall permeability. Additionally, we developed genomic mutant strains, Msm-Y252A and Msm-Q255A, featuring targeted point mutations in the substrate-specific site (S-site) of the RNR loop domain, which determines NDP reduction specificity. The Msm-Y252A displayed a 1.9-fold decrease in dATP and increases in dGTP (1.6-fold), dTTP (9.0-fold), and dCTP (1.3-fold). In contrast, Msm-Q255A exhibited elevated intracellular levels of dGTP (1.6-fold), dTTP (6.1-fold), and dATP (1.5-fold), while dCTP levels remained unchanged. Neither the NrdE knockdown strain nor the S-site mutants exhibited direct resistance development; however, they both showed genomic instability, enhancing the emergence of rifampicin-resistant mutants, even with a 70-fold and a 25-fold increase in mutation frequency for Msm-Y252A and Msm-Q255A, respectively. This study demonstrates that NrdE is integral to Mycobacterium survival and genomic stability and that its RNR dysfunction creates a mutagenic environment under selective pressure, indirectly contributes to the development of drug resistance, positioning NrdE as an effective target for therapeutic strategies and a valuable molecular marker for early detection of drug-resistant Mtb.

## Introduction

Tuberculosis (TB), caused by *Mycobacterium tuberculosis* (*Mtb*), is highly infectious. Since the introduction of antibiotics in the 1940s, the rise of multidrug-resistant (MDR-TB) and extensively drug-resistant TB (XDR-TB) has posed significant challenges for TB prevention and control [1]. Unlike many bacteria, *Mtb* does not undergo plasmid-mediated horizontal gene transfer and cannot acquire drug-resistance genes from other species; instead, all drug resistance in *Mtb* arises from genetic mutations [2,3]. Therefore, *Mtb*'s mutation capacity is a critical factor in its development of drug resistance.

Mutations that arise from nucleotide mismatches during DNA replication are a primary cause of mutagenesis. High-fidelity DNA polymerases replicate DNA with error rates as low as 10^−^⁶per base pair, ensuring accuracy. In *Mtb*, mutations in the α subunit (DnaE) and the 3'−5’ exonuclease ϵ subunit (DnaQ) of DNA polymerase impair proofreading activity, leading to elevated mutation rates and accelerated drug resistance evolution [4,5]. Moreover, DNA replication fidelity also depends on the correct balance and overall concentration of deoxynucleoside-5′-triphosphates (dNTPs) [6,7,8].

Ribonucleotide reductase (RNR) is the key enzyme responsible for synthesizing and regulating dNTPs by reducing ribonucleoside diphosphates (NDPs) – ADP, GDP, CDP, and UDP – to their corresponding deoxynucleotides [9,10,11]. RNR exists in several classes based on its structure and the metal ion cofactors required for its activity. These include class I, class II, and class III RNRs. Class I RNRs, which are the most studied and are used by nearly all aerobic organisms, are typically tetramers consisting of a dimer of large subunits (R1) and a dimer of small subunits (R2). The large subunit contains a catalytic site (C-site) and two allosteric regulatory sites: the substrate specificity site (S-site) and the activity site (A-site). In contrast, class II and class III RNRs have distinct structures and mechanisms of action, with class II RNRs requiring a diferric cluster and class III RNRs depending on a radical generated by an iron-sulfur cluster. Notably, in several bacteria such as *Mtb*, *Bacillus subtilis*, and *Streptococcus pneumoniae*, their class Iβ RNRs lack the ATP-cone at the A-site, a feature that distinguishes their regulation from that of class I RNRs in other organisms. Therefore, their regulation of RNR activity relies more heavily on the allosteric effects mediated by the S-site [12]. The S-site, located at the interface of the R1 dimer, receives input from a flexible loop domain contributed by each R1 monomer (loop1 and loop2) [13–15]. When specific effectors bind to the S-site, conformational changes are transmitted to the C-site, determining which NDP substrate is reduced. ATP binding at the S-site favors CDP reduction, dATP promotes UDP reduction, while dTTP and dGTP binding facilitate the reduction of GDP and ADP, respectively, thereby ensuring stable dNTP levels within the cell [16]. Thus, RNR R1 subunit plays an essential role in maintaining dNTP concentration and ratio, supporting DNA replication fidelity.

In *Mtb*, the R1 and R2 subunits of RNR are essential genes, encoded by *nrdE* (*Rv3051c*) and *nrdF* (*Rv3048c*), respectively. However, no studies have yet explored the potential relationship between RNR and genomic stability or drug resistance development in this organism. By analysing mutations in RNR-encoding genes in the whole-genome sequences of 1,393 MDR-TB strains and 978 drug-sensitive *Mtb* strains from public databases, an amino acid mutation was identified within the functional loop1 domain of S-site of NrdE in MDR-TB (Table S1 and S2). It has been reported that any mutation in the S-site may increase the mutation rate in bacteria [17,18], and we wondered whether there was a possible link between NrdE mutations and *Mtb* resistance.

In this study, we further constructed NrdE knockdown strains in both *Mtb* and *Mycolicibacterium smegmatis* (*Msm*). We found that NrdE is crucial for maintaining genomic stability in mycobacteria. Its RNR dysfunction can disrupt oxidative homeostasis and dNTP pool balance and increase DNA replication errors, ultimately leading to higher bacterial genome mutation rates and facilitating drug resistance acquisition.

## Methods

### Bacterial strains, plasmids, and culture conditions

*Msm* and *Mtb* were cultured in 7H9 broth (Difco,USA) with oleic acid – albumin – dextrose – catalase (OADC), 0.05% Tween-80, and 0.2% glycerin or on 7H10-OADC agar medium (Difco, USA) with 0.5% glycerin. When necessary, ATC (100 ng/mL; MedChemExpress, USA) was added. *E. coli* DH5α was used for genetic manipulation of DNA and was grown in Luria–Bertani (LB) medium (Sangon, China). Antibiotics were added at the following concentrations: kanamycin (Sangon, China), 50 μg/mL for *E. coli*, 30 μg/mL for *Msm*, and 20 μg/mL for *Mtb*; hygromycin (Sangon, China), 150 μg/mL for *E. coli*, 75 μg/mL for *Msm*, and 50 μg/mL for *Mtb*. All cultures were incubated at 37 °C.

Two induced NrdE knockdown strains, *Msm-Ms2299* KD and *Mtb*-*Rv3051c* KD, were constructed using the clustered regularly interspaced short palindromic repeats interference (CRISPRi) technique [19]. SgRNAs were designed based on their respective gene sequences and cloned into the CRISPRi backbone, i.e. PLJR-962 for *Msm* and PLJR-965 for *Mtb*. The PLJR backbone includes the TetR repressor, which suppresses the expression of dCas9 and sgRNA. Upon addition of ATC, it binds to TetR, inducing a conformational change that causes TetR to dissociate from the promoter, thereby initiating CRISPRi-mediated gene silencing [20]. *Msm*-*Ms2299* KD and *Mtb*-*Rv3051c* KD were cultured to logarithmic growth phase (OD_600 _= 0.6) in 7H9-OADC containing 100 ng/mL ATC for ensuring the activation of CRISPRi and effective gene silencing.

For constructing CRISPRi-resistant complementation strains, gene fragments of *Ms2299* and *Rv3051c* were amplified from *Msm* and *Mtb*, respectively, and ligated into the pMV361 plasmid. To prevent sgRNA targeting, the allele protospacer adjacent motif on pMV361-derived constructs was synonymously mutated (F505F, (TTC to TTT) for *Ms2299* and F509F (TTC to TTT) for *Rv3051c*) using the KOD-Plus-Mutagenesis Kit (TOYOBO, Japan). These constructs were introduced into knockdown strains via electroporation, resulting in CRISPR-resistant complementation strains *Ms2299* KD + *Ms2299*, *Ms2299* KD + *Rv3051c*, and *Rv3051c* KD + *Rv3051c*. All primers used are listed in Table S3.

The *Y252A* and *Q255A* mutant strains of *Msm* were constructed with a CRISPR-Cas12a system [21]. In brief, the system utilizes homologous repair mechanisms by designing sgRNAs to target the gene of interest, thereby inducing double-strand breaks (DSBs) at precise genomic locations. Following the DSB induction, a repair template (such as a single-stranded oligonucleotide containing the desired mutation) is supplied, facilitating the incorporation of the point mutation through homology-directed repair (Table S3).

### Observation of mycobacterial phenotype

Strains were cultured to log phase (OD_600 _= 0.5-1.0), adjusted to an OD_600_ of 0.2, and reinoculated in fresh 7H9-OADC medium with ATC at a 1:100 dilution. Cultures were incubated at 37 °C through the entire growth phase. Samples were collected at the same growth stage, and OD_600_ values were measured every 2 h for *Msm* strains or daily for *Mtb* strains after growth initiation. Experiments were performed in triplicates, and average values were used to generate growth curves.

Log-phase *Msm* strains were spotted in a 10-fold serial dilution on 7H10 containing ATC and incubated at 37 °C for 4–8 days. Colony morphology was observed and recorded under a stereomicroscope (Axio Zoom.V16, Zeiss, Germany).

### Minimal inhibit concentration (MIC) determination and survival curves

The MIC of drugs was determined as previously described [22]. For MIC determination of knockdown and complementation strains, ATC was also present during incubation with antibiotics. After 3 days of incubation at 37 °C, the lowest concentration that prevented visible growth of *Msm* was defined as the MIC.

Strains were initially grown in media containing ATC until reaching the logarithmic growth phase (OD_600 _= 0.6), at which point knockdown of NrdE was determined to be established, and the phenotype was stable. After washing once with fresh medium without ATC, the bacteria were exposed to different antibiotics and CFUs were counted at different time points. Both drug exposure process and CFU counting were performed in ATC-free medium. The percentage of CFUs recovered was determined relative to an untreated control sampled at the time antibiotics were added. Each experiment was repeated at least three times.

### RT-qPCR and RNA-Seq

Total RNA extraction followed the TRIZOL method (Invitrogen, USA) [23]. Using 1 μg of this RNA, cDNA was synthesized via the HiScript II one-step RT–PCR kit (Dye Plus) (Vazyme, China). For qPCR, each reaction was run in triplicate using the Taq Pro universal SYBR qPCR master mix (Vazyme, China) to amplify cDNA. The relative transcriptional levels of genes were quantified by the 2^-ΔΔCT^ method, with 16S rRNA as the reference. Details of qPCR primers are in Table S3.

RNA was processed by Majorbio Bio-Pharm Technology Co., Ltd. (Shanghai, China). Transcriptome libraries were prepared with a TruSeqTM RNA Sample Preparation Kit (Illumina, USA), and sequences were annotated through Gene Ontology (GO) for functional genes identification and metabolic pathways analysis. Raw counts across samples were normalized using the TMM method, and the expression difference was assessed via the Majorbio Cloud Platform.

### Mycolic acid assay

Log-phase *Msm* (50 mL) was resuspended in 720 μL of distilled water and 1,080 μL of 25% tetrabutylammonium hydroxide solution (Aladdin, China) and incubated at 100 °C for 7 h. After cooling, 2 mL of distilled water, 3 mL of dichloromethane, and 300 μL of iodomethane were added, shaken for 1 h, and the organic phase was left to dry overnight. The samples were treated with 1.5 mL of anhydrous ether, ultrasonicated (power 30%, one minute), and the supernatant was transferred to empty tubes and dried to obtain a yellow oily substance. Samples were dissolved in a solvent mixture (chloroform: methyl alcohol = 2: 1) to a concentration of 33 μg/μL, and 6 μL (approximately 200 μg) was spotted for further analysis.

The developing agent (n-hexane: acetate = 19: 1) was added to a chromatography tank and pre-saturated for 20 min. The chromatography plate was placed into the tank and developed until the solvent front reached the finish line. The plate was removed, air-dried, and redeveloped. This process was repeated three times. A 5% phosphomolybdic acid-ethanol solution was sprayed onto the plate, which was then dried and developed using a heat gun at 400 °C.

### Ethidium bromide (EB) accumulation

*S*trains were growth in media containing ATC to OD_600 _= 0.6, washed twice with 0.4% glucose and adjusted to an OD_600_ of 0.6. A total of 200 μL of the suspension cells were added in triplicate to a 96-well black fluoroplate, and EB was added to final concentrations (2 μg/mL). Accumulation of the dyes was measured with excitation at 545 nm and emission at 600 nm for EB.

### Analysis of frequencies of mutation to RIF^R^

Strains were harvested after growth to OD_600 _= 0.6, and resuspended in 100 μL of PBS, and plated on 7H10 agar plates containing 100 μg/mL RIF. Cell counts were determined by plating dilutions. The RIF^R^ mutation frequency was calculated by dividing the number of RIF-resistant colonies on a RIF plate by the counts of the total viable cells plated.

### Sequencing of rpoB mutations

RIF-resistant colonies were collected and inoculated into 200 μL of 7H9-OADC containing 100 μg/mL RIF at 37 °C for 6–7 days. The *rpoB* motif cluster was amplified using bacteria as a template. PCR products were sent to Sangon Biotech Co., Ltd (China) for sequencing.

### dNTP pool measurements

The measurement of dNTP pools is based on previous methods, with some modifications [24,25]. Bacterial samples (100 mL) were resuspended in 15 mL of 60% methanol at – 20°C for overnight. The cell extract was freeze-dried, resuspended in 0.5 mL of distilled H_2_O, and further extracted with 0.5 mL of chloroform. The aqueous phase was collected, freeze-dried again, and finally resuspended in 0.2 mL of distilled water. The nucleotide library was analysed by reversed-phase High-Performance Liquid Chromatography (HPLC) with UV detection at 254 nm using an Agilent 1260 Infinity HPLC system. Commercial solutions of four dNTPs, ADP and ATP (Aladdin, China) were used as standard reagents.

Nucleotides were separated on a Hypersil GOLD C18 column (4.6 × 250 mm, 5 μm; Thermo, USA) at a flow rate of 0.8 mL/min, with a linear gradient of 40:60:0 (buffer A:B:C) run to 0:70:30 at 30 min. Buffer A consisted of 5 mM t-butyl ammonium phosphate (Thermo, USA), 10 mM KH_2_PO_4_, and 0.25% methanol adjusted to pH 6.9. Buffer B consisted of 5 mM t-butyl ammonium phosphate, 50 mM KH_2_PO_4_, and 30% methanol (pH 7.0). Buffer C was acetonitrile.

Since ATP levels are consistently stable in cells and unaffected by growth rates, raw dNTP values (height, mAU) were normalized using ATP as an internal standard [26]. Specifically, the normalization factor for each sample was calculated by dividing the ATP value in each sample by the ATP value of WT sample. Subsequently, the raw dNTP values (dATP, dCTP, dTTP, and dGTP) were normalized by dividing each by its respective normalization factor.

The total quantity of each nucleotide was determined by comparing the HPLC peak areas to known standard curves recovered from the same chromatography columns. To account for potential losses during the multi-step extraction process, we evaluated the extraction efficiency using standards. Finally, the values were adjusted for losses during preparation process and the number of cells used to calculate dNTP concentrations in picomoles per 10^7^ cells.

### ROS determination and NAD^+^/NADH ratio assay

*Msm* was stained with 10 μM Dihydroethidium (DHE; Thermo, USA) for 30 min, and fluorescence intensity was measured with excitation at 485 nm and emission at 620 nm. CFU counting was conducted on the bacterial suspension to quantify relative ROS.

*Msm* was lysed using ultrasonication, and the lysate was divided into two tubes for NADH and NAD^+^ measurements with the Amplite Colorimetric NAD^+^/NADH Ratio Assay Kit (AAT Bioquest, USA). Absorbance was monitored using an absorbance plate reader (Biotek, USA) at 460 nm.

### Measurement of catalase (CAT) and superoxide dismutase (SOD) activity

The enzyme activity of CAT and SOD was measured using assay kits (CATM-W48-N (1620) for CAT and SODW-W96-N (1733) for SOD; Mlbio, China). CAT activity was determined by the ammonium molybdate colorimetric method, where the reduction in absorbance was measured at 405 nm following the reaction between H_2_O₂ and ammonium molybdate. SOD activity was quantified using the WST-8 method, where the reduction of WST-8 to a formazan dye is proportional to the enzyme activity, and the absorbance was measured at 450 nm.

### Flow cytometry-based membrane potential detection and DNA fragmentation assays

Membrane potential was detected using a BacLight Bacterial Membrane Potential Kit (Thermo, USA). *Msm* was diluted to 1 × 10^6^ cells/mL, then depolarized with 30 mM DiOC_2_ for 30 min. Membrane potential was determined by the ratio of red to green fluorescence intensities by an LSR Fortessa flow cytometer (BD, USA).

DNA strand breaks in *Msm* strains were assessed by TUNEL using an in-situ cell death detection kit (Roche, Switzerland). PBS-washed bacteria were permeabilized and stained with a TUNEL reaction mix as recommended by the manufacturer, and labelled for 1 h at 37 °C in the dark. After labelling, cells were rinsed with PBS and analysed by flow cytometry.

Flow Cytometry data acquisition utilized BD FACS Diva software (version 8.0.1), with instrument settings as follows: forward scatter (FSC) at 200 V log, side scatter (SSC) at 150 V log, and an SSC threshold of 1,000 V. Samples were excited with a 488-nm laser, and emissions were captured by 505 long pass and 525/50 band pass filters. Each sample was measured across 10,000 events.

### Mice infection

C57BL/6 mice, aged 6–8 weeks, were infected intravenously via the tail with 4 × 10^7^ CFU of *Msm, Msm-Y252A* and *Msm-Q255A*, respectively*.* At 5-, 12 – and 18-days post-infection, the mice were sacrificed to count CFUs in the lungs and spleens. One half of each lung and spleen was fixed in 4% neutral-buffered paraformaldehyde for 24 h. The tissues were subsequently embedded in paraffin and stained with hematoxylin and eosin following standard protocols. The protocol used in animal experiments was approved by The Animal Care and Use Committee of School of Life of Fudan University.

### Statistical analysis

For statistical analysis, ANOVA and an unpaired two-tailed Student’s *t*-test were performed with GraphPad Prism 8.0, considering *p* < 0.05 as statistical significance.

## Results

### Knockdown of nrdE severely inhibits bacterial growth

**Figure 1.**
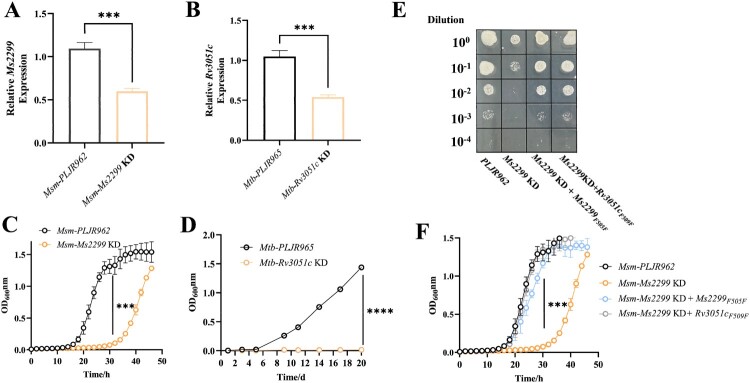
Phenotypes of NrdE knockdown and complementation strains. (A) qPCR analysis of *nrdE* (*Ms2299*) expression in *Msm*-*PLJR962* and *Msm*-*Ms2299* KD. (B) qPCR analysis of *nrdE* (*Rv3051c*) expression in *Mtb*-*PLJR965* and *Mtb*-*Rv3051c* KD. (C, D) Growth curves of *Msm* and *Mtb* strains in 7H9-OADC medium with ATC. (E) Ten-fold serial dilutions of *Msm* complementation strains were spotted on Middlebrook 7H10 with ATC. (F) Growth curves of complementation strains in 7H9-OADC medium with ATC. Data represent the mean (*n* = 3) ± SD. ***, *p* < 0.001; ****, *p* < 0.0001.

NrdE, the large subunit of RNR (R1), is essential for bacterial growth [27] and highly conserved, with the amino acid sequences of *Mtb* and *Msm* showing 92% identity and 96% similarity. We generated inducible NrdE knockdown strains in both *Mtb* H37Ra and *Msm* using the CRISPRi system. RT-qPCR results confirmed that *nrdE* in *Msm*-*Ms2299* KD and *Mtb*-*Rv3051c* KD was significantly downregulated compared to the control strain with an empty plasmid (Figure 1A, B). NrdE knockdown delayed *Msm* growth from the lag phase, with *Msm*-*Ms2299* KD reaching the stationary phase after 46 h, compared to *Msm*-*PLJR962*, which reached the stationary phase after 30 h (Figure 1C). Furthermore, the mutant cells (8.5 μm) were significantly longer than the wild-type (WT) cells (4.7 μm) (Figure S1). In *Mtb*, NrdE knockdown resulted in even more pronounced growth defects, with cells remaining in the lag phase for 20 days (Figure 1D). These findings collectively underscore the essential role of NrdE in bacterial growth.

The Cas9-sgRNA complex targets the DNA site, blocking *nrdE* transcription and elongation, thereby reducing its expression. To construct CRISPRi-resistant complement strains, synonymous mutations (F505F for *Ms2299*, F509F for *Rv3051c*) were introduced in the protospacer adjacent motif (PAM) of the complementation alleles to prevent sgRNA targeting. Results showed that growth inhibition in *Msm* due to *Ms2299* knockdown could be restored by introducing *Ms2299_F505F_*, while the introduction of *Ms2299_WT_* did not (Figure S2A, B). Thus, in all further studies, complementation strains refer to PAMs with synonymous mutations that confer CRISPRi resistance. Similarly, introducing *Rv3051c_F509F_* also rescued the growth defects of *Msm-Ms2299* KD (Figure 1E, F), indicating that *Rv3051c* (the *nrdE* homolog in *Mtb*) functionally complements *Ms2299* in *Msm*.

### Knockdown of nrdE increases sensitivity to multiple antibiotics and stresses

**Figure 2.**
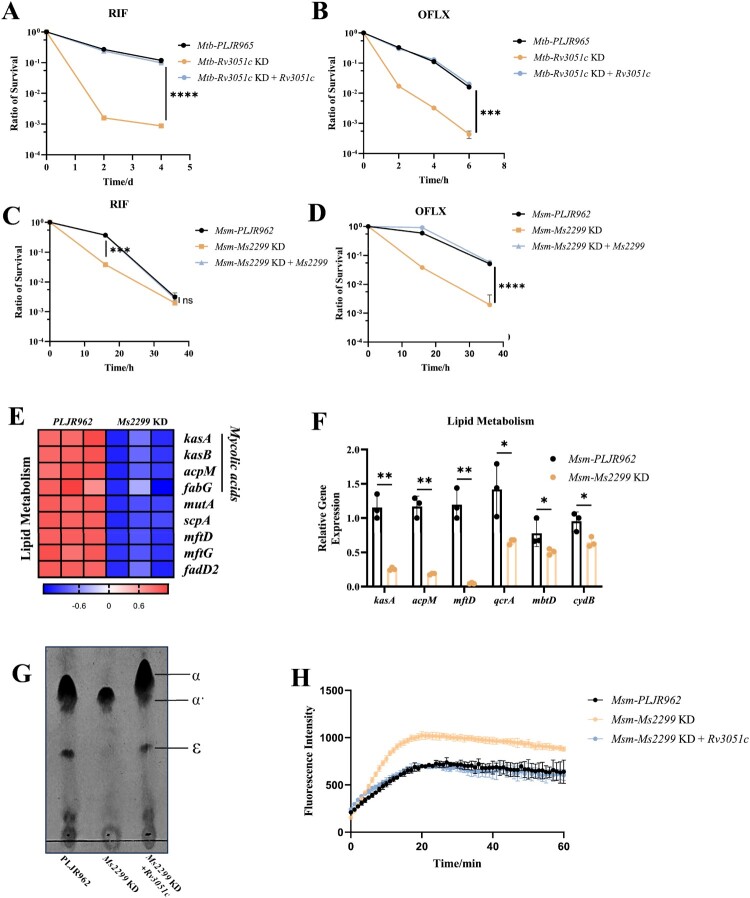
NrdE knockdown increases antibiotic susceptibility by enhancing cell wall permeability. (A, B) Survival of *Mtb* strains exposed to lethal concentrations of antibiotics (10× MIC), including RIF (A) and OFLX (B). (C, D) Survival of *Msm* strains (complemented with *Ms2299*) exposed to lethal antibiotics (10× MIC), including RIF (C) and OFLX (D). (E, F) Differential expression of lipid metabolism genes in *Msm*-*Ms2299* KD, as shown by RNA-seq (*p* < 0.05 and |log_2_FC| ≥ 1) and qPCR. (G) TLC analysis of methyl esters of mycolic acids from log-phase *Msm* strains. Methyl esters were separated in n-hexane/ethyl acetate (95:5; three runs). α: α-mycolate; α': α'-mycolate; ϵ: epoxy-mycolate. (H) Log-phase cultures of *Msm* strains incubated in PBS with 0.04% glucose and 2 μg/mL EB; fluorescence was measured every minute for one hour. All experiments were performed in triplicate with similar results. Data represent the mean (*n* = 3) ± SD. *ns*, not significant; *, *p* < 0.05, **, *p* < 0.01, ***, *p* < 0.001, ****, *p* < 0.0001.

Next, we evaluated the MICs of various antibiotics, including INH, RIF, ofloxacin (OFLX), streptomycin (S), capreomycin (CAP), clofazimine (CFZ), erythromycin (ERY), and fusidic acid (FA). Compared to *Msm*-*PLJR962* and the complement strains, *Msm*-*Ms2299* KD was slightly more susceptible to these antibiotics, with approximately two-fold lower MICs (Table 1). The survival rates of *Msm*-*Ms2299* KD and *Mtb*-*Rv3051c* KD were significantly reduced at lethal antibiotic concentrations (10-fold MIC of RIF and OFLX) compared to the *WT* and complement strains (Figure 2A–D, Figure S3A–D).

**Table 1.** MIC of various antibiotics for Msm-PLJR962, Msm-Ms2299 KD, Msm-Ms2299 KD + Rv3051c and Msm-Ms2299 KD + Ms2299. INH, isoniazid; RIF, rifampicin; OFLX, ofloxacin; CFZ, clofazimine; STR, streptomycin; ERY, erythromycin; FA, fusidic acid; CAP, capreomycin.

Strain	MIC(μg/ml)							
	INH	RIF	OFLX	CFZ	STR	ERY	FA	CAP
*Msm*-*PLJR962*	25	18.7	0.625	5	0.625	40	128	3.12
*Msm*-*Ms2299* KD	12.5	9.3	0.312	1.25	0.625	10	64	1.55
*Msm*-*Ms2299* KD + *Rv3051c*	25	18.7	0.625	2.5	0.625	20	128	3.12
*Msm*-*Ms2299* KD + *Ms2299*	25	18.7	0.625	2.5	0.625	10	128	3.12

We also assessed the viability of the NrdE knockdown strain under various environmental stresses, including low pH, oxidative stress, heat shock, and the presence of a surfactant (0.05% SDS). The results showed that *Msm*-*Ms2299* KD survival decreased by approximately 1–2 log CFU under these conditions compared to *Msm*-*PLJR962* (Figure S3E-H). These findings indicate that NrdE knockdown heightens mycobacterial sensitivity to unfavorable environmental conditions.

### Effects of nrdE knockdown on mycobacterial lipid metabolism, and cell wall permeability

To explore the physiological role of NrdE in mycobacteria, we performed transcriptional profiling, which revealed that 617 genes were differentially expressed (*p* < 0.01, |log_2_FC| ≥ 2) in *Msm*-*Ms2299* KD, with 331 genes upregulated and 286 downregulated (Figure S4A). GO enrichment analysis indicated significant enrichment of genes involved in cellular metabolism, response to stimuli, and redox pathways (Figure S4B). Notably, lipid biosynthesis genes, including fatty acid synthase II (FAS II)-related genes (*kasA*, *kasB*, *acpM*, and *fabG*), were downregulated by approximately four-fold in *Msm*-*Ms2299* KD (Figure 2E). These transcriptional changes were validated by RT-qPCR (Figure 2F). FAS II is crucial for mycolic acid synthesis, a key component of the mycobacterial cell wall [28]. By using thin-layer chromatography (TLC), we assessed the levels of mycolic acid subtypes produced, including α-mycolate (α), α'-mycolate (α’), and epoxy-mycolate (ϵ). The results showed that the levels of mycolic acid in knockdown strain produced was significantly reduced, especially for the α and ϵ subtypes compared to the *WT* and complement strains. (Figure 2G). As mycolic acid reduction increases cell wall permeability [29], EB accumulation assays indicated significantly faster and higher pore accumulation in the knockdown strain than in the control (Figure 2H, Figure S3I). Given that mycolic acid synthesis primarily occurs during the active growth phase of bacteria, we hypothesize that growth-impaired NrdE knockdown strain would shift into an energy-saving growth mode by down-regulating lipid metabolism and reducing mycolic acid synthesis, which indirectly results in an increase in cell wall permeability and sensitivity of Mycobacteria to a variety of antibiotics and environmental stresses.

### Knockdown of nrdE disrupts the redox balance and increases the mutation rate of mycobacteria

**Figure 3.**
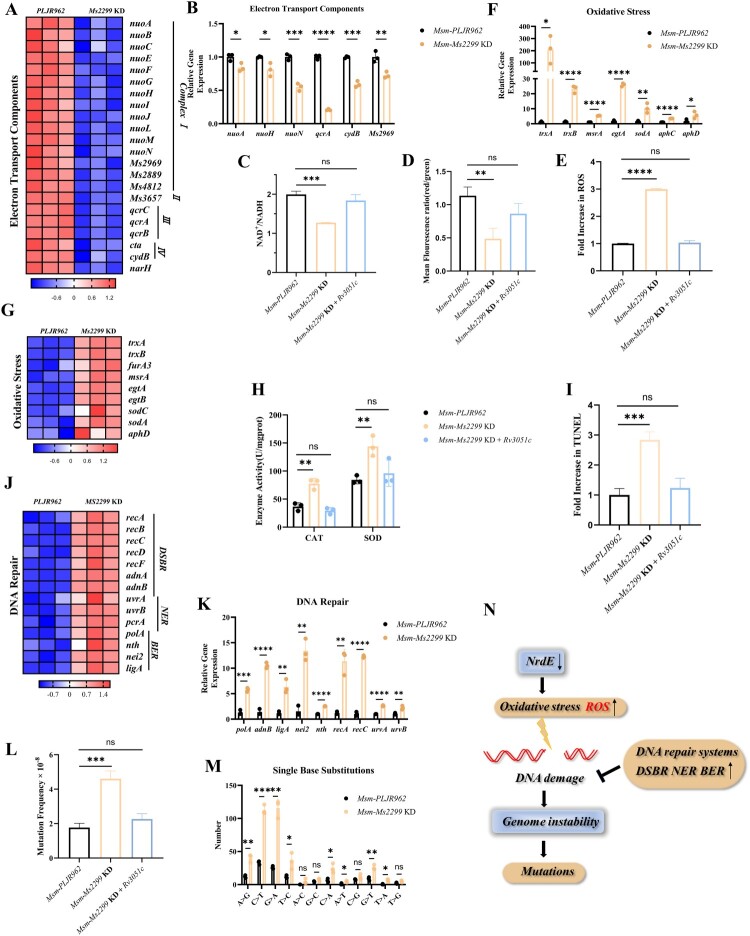
NrdE knockdown disrupts redox balance and elevates mutation rate in mycobacteria. (A-B, F-G) Differential expression of genes related to electron transport components and oxidative stress in *Msm*-*Ms2299* KD*,* as determined by RNA-seq (*p* < 0.05 and |log_2_FC| ≥ 1) and qPCR. (C) Comparison of NAD^+^/NADH ratios, (D) membrane potential, (E) ROS levels, and (H) CAT and SOD activity in *Msm*-*PLJR962*, *Msm*-*Ms2299* KD, and *Msm*-*Ms2299* KD* + Rv3051c*. (I) Flow cytometry analysis of DNA double-strand breaks in strains stained with TUNEL. (J, K) Differential expression of DNA repair genes in *Msm*-*Ms2299* KD, shown by RNA-seq (*p* < 0.05 and |log_2_FC| ≥ 1) and qPCR. (L) Frequency of RIF^R^ mutations in *Msm*-*PLJR962*, *Msm*-*Ms2299* KD, and *Msm*-*Ms2299* KD* + Rv3051c*. (M) Mutation spectra in *Msm*-*PLJR962* and *Msm*-*Ms2299* KD. (N) Schematic representation: NrdE knockdown induces oxidative stress and ROS accumulation, leading to DNA fragmentation, increased mutation rates, and compromised genomic stability. Data represent the mean (*n* = 3) ± SD. *ns*, not significant; *, *p* < 0.05, **, *p* < 0.01, ***, *p* < 0.001, ****, *p* < 0.0001.

Transcriptome sequencing and qRT-PCR results revealed that NrdE knockdown significantly down-regulated several genes associated with the respiratory chain (electron transport complexes I-IV) (Figure 3A-B), suggesting that NrdE knockdown affects cell growth and may in turn reduce respiratory chain activity. Consequently, the NAD^+^/NADH ratio and membrane potential in *Msm-Ms2299* KD were significantly lower than in the control (Figure 3C-D, Figure S5A). This reduction in respiratory chain function hinders the extraction of electrons from NADH and the translocation of protons from the cytosol to the intermembrane space, resulting in decreased membrane potential and altered NADH levels [30].

Down-regulation of respiration disrupts redox homeostasis, as evidenced by the oxidation of NADH by respiratory complex I [31] (Figure 3A). Consistently, reactive oxygen species (ROS) levels were observed to have an approximately 2-fold increase in the knockdown strain compared to the control (Figure 3E, Figure S5B). Simultaneously, knockdown of NrdE led to up-regulation of redox-related genes, including *trxB* (thioredoxin reductase), *sodA* (superoxide dismutase), and *msrA* (methionine sulfoxide reductase A) (Figure 3F-G). Increased catalase and SOD enzyme activities further supports this finding (Figure 3H), confirming that disruption of redox homeostasis leads to compensatory changes in gene expression to attenuate oxidative stress [30].

Elevated intracellular ROS is a primary cause of DNA damage and genetic mutations [32–35]. TUNEL assays confirmed a 3-fold increase in DNA damage in the NrdE knockdown strain relative to the control (Figure 3I). Cells responded to this DNA damage by upregulating four types of DNA repair genes: nucleotide excision repair (NER), mismatch repair (MMR), double-strand break repair (DSBR), and base excision repair (BER) (Figure 3J–K) [36].

To investigate the impact of reduced RNR activity on bacterial genome stability, the spontaneous mutation frequency for RIF-resistance in *Msm* was assessed. Compared to *Msm-PLJR962* and *Msm-Ms2299* KD* + Rv3051c*, the RIF spontaneous mutation frequency in *Msm-Ms2299* KD increased by approximately 2-fold (Figure 3L, Figure S5C). Additionally, the number of single base substitutions (SBSs) in *Msm-Ms2299* KD was significantly higher (461.7 ± 35.59 *vs.* 149 ± 5.71, *p* = 0.0003), with a mutation spectrum dominated by CG > TA and TA > CG transitions and CG > AT transversions (Figure 3M). Overall, NrdE knockdown induces oxidative stress and ROS accumulation, leading to DNA damage and genomic instability (Figure 3N).

### Mutants y252a and q255a in the S-site of R1 increase the mutation rate by disrupting the dNTP pool balance

**Figure 4.**
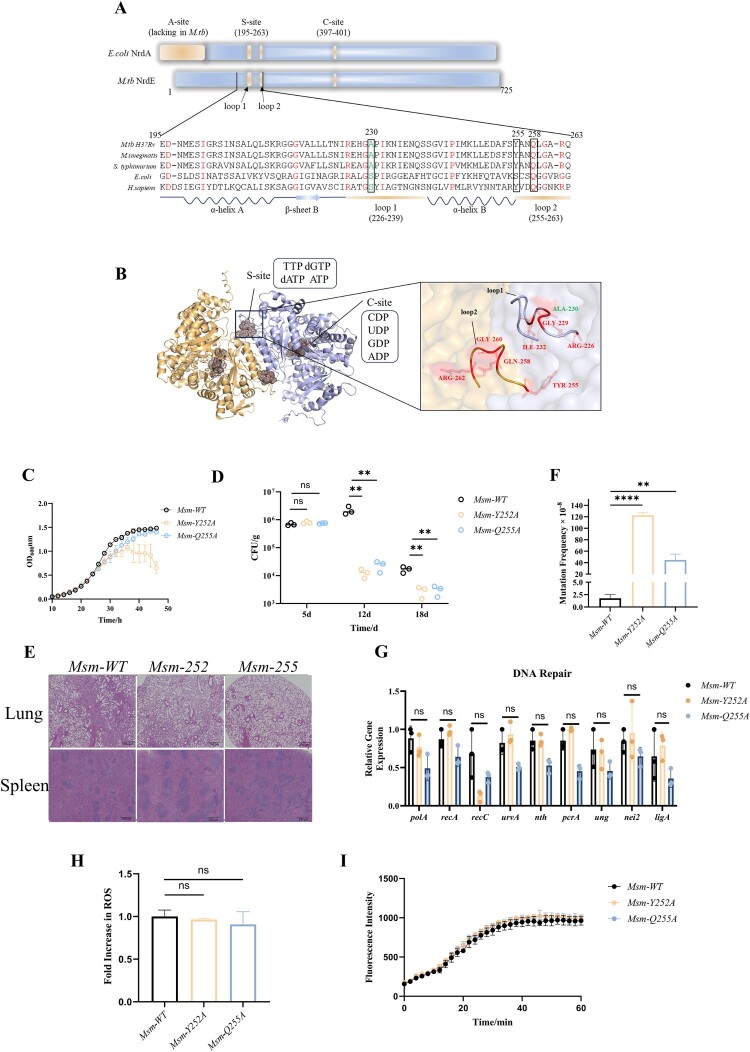
Functional and Phenotypic Analysis of S-site mutants *Msm*-*Y252A* and *Msm*-*Q255A*. (A) Schematic diagram of the primary structure of R1 in various species*.* The active domain showed in yellow and amino acid sequences of S-sites were showed below. Completely conserved and clinical isolates-identified amino acids were highlighted in red and green respectively. (Amino acid sequence number based on *Mtb. Y252 and Q255 in Msm, are homologous to Y255 and Q258 in Mtb, respectively.*) (B) Predicted tertiary structure of *Mtb* R1 by AlphaFold2, with a magnified view of the S-site; conserved and clinically identified amino acids are highlighted in red and green, respectively. (C) Growth curves of *Msm*-*WT*, *Msm*-*Y252A*, and *Msm*-*Q255A* strains. (D) CFU counts in the spleens of mice infected with *Msm*-*WT*, *Msm*-*Y252A*, and *Msm*-*Q255A*. C57BL/6 mice were intravenously via the tail challenged with 4.0 × 10^7^ CFU of *Msm* in 200 µL PBS. *Msm* load was quantified by CFU on days 5, 12, and 18 post-infection (n = 3). (E) Histopathology of lung and spleen tissues of mice infected with *Msm*-*WT*, *Msm*-*Y252A*, and *Msm*-*Q255A*. (F) Spontaneous RIF^R^ mutation frequencies in *Msm*-*WT*, *Msm*-*Y252A*, and *Msm*-*Q255A*. (G) qPCR analysis of DNA repair-related genes expression in *Msm*-*WT*, *Msm*-*Y252A*, and *Msm*-*Q255A*. (H) Comparison of ROS in *Msm*-*WT*, *Msm*-*Y252A* and Msm-*Q255A*. (I) Log-phase cultures of *Msm* strains incubated in PBS with 0.04% glucose and 2 μg/mL EB; fluorescence was measured every two minutes for one hour. Data represent the mean (*n* = 3) ± SD. *ns*, not significant; *, *p* < 0.05, **, *p* < 0.01, ***, *p* < 0.001, ****, *p* < 0.0001.

In addition to total dNTP concentration, the relative concentrations of the four dNTPs (dATP, dTTP, dGTP, dCTP) are critical for maintaining DNA synthesis fidelity [37]. The substrate reduced at the RNR C-site is determined by allosteric regulation at the S-site of R1 [14,15]. A subset of clinical drug-resistant *Mtb* isolates (Ala230Gly, 0.64%) harboured NrdE mutations in the loop1 of R1 S-site (Figure 4A and Table S1). AlphaFold2 predictions suggested that each R1 monomer forms a homodimer, using flexible loop1 and loop2 to create the S-site (Figure 4B). We hypothesized that specific amino acid substitutions in the loop domain could alter RNR functional regulation, affecting dNTP concentrations, increasing DNA replication errors, and promoting drug resistance.

To test this hypothesis, we attempted to introduce point mutations into the *Msm* genome by targeting conserved amino acids in the loop1 and loop2, as well as targeting identified amino acid of S-site in clinical isolates (highlighted in Figure 4A–B), using the CRISPR-Cas12a system. Unfortunately, due to the limited efficiency of genomic-targeted mutagenesis, we only succeeded in generating two mutations in the loop2 domain of S-site: *Msm-Y252A* and *Msm-Q255A*. *Msm-Y252A* displayed a division-impaired phenotype with growth retardation in the late log phase, whereas *Msm-Q255A* exhibited growth comparable to the control (Figure 4C). In addition, there was no significant difference in the survival of the two mutations and the wild strain *in vivo* at 5 days post-infection. However, at 12 and 18 days post-infection in mice, the bacterial loads in spleen tissues of the Y252A and Q255A mutant strains were significantly decreased compared to WT (Figure 4D). In addition, histopathology of the lungs and spleen at 18 days post-infection showed more severe damage in the WT group (Figure 4E). These findings suggest that the Y252A and Q255A point mutations undermine bacterial survival *in vivo*.

Furthermore, dNTP levels in the mutant strains and *WT* were measured by HPLC. In *Msm-Y252A*, dCTP, dGTP, and dTTP levels increased by 1.3-, 1.6-, and 9.0-fold, respectively, while dATP decreased by 1.9 – fold. In *Msm-Q255A*, dGTP, dTTP, and dATP increased by 1.6-, 6.1-, and 1.5-fold, respectively, with unchanged dCTP (Table 2). Both mutants exhibited dramatically increased RIF spontaneous mutation frequency, with 70-fold and 25-fold increases for *Msm-Y252A* and *Msm-Q255A,* respectively, compared to the *WT* mutation frequency (1.76 × 10^−8^) (Figure 4F). However, there were no significant changes in the expression of DNA repair genes, intracellular ROS levels, and cell wall permeability in *Msm-Y252A* and *Msm-Q255A* strains compared to the wild strain (Figure 4G–I). Thus, imbalances in dNTP pool caused by mutants in the S-site of loop domain, even modest changes, can affect DNA replication fidelity [38], destabilizing the genome [7,8], and ultimately increasing mutation rates, consistent with those found in *E.coli* and yeast [26,39].

**Table 2.** HPLC analysis of deoxyribonucleotide pools in Msm*.*

	Peak area (mAU·min) ^a^			dNTP content (pmol/10^7^ cell) ^b^		
	WT	Y252A	Q255A	WT	Y252A	Q255A
dATP	81.90 ± 13.36	42.42 ± 1.04	118.94 ± 7.40	1.075 ± 0.175	0.557 ± 0.014	1.561 ± 0.097
dCTP	148.66 ± 19.25	187.82 ± 9.07	144.01 ± 12.35	1.951 ± 0.253	2.465 ± 0.119	1.890 ± 0.162
dTTP	14.13 ± 0.45	132.15 ± 17.29	86.75 ± 6.84	2.781 ± 0.088	26.019 ± 3.405	17.079 ± 1.346
dGTP	38.82 ± 3.47	60.78 ± 2.74	60.97 ± 3.58	0.510 ± 0.046	0.798 ± 0.036	0.800 ± 0.047

^a^ To correct for minor differences between samples, the dNTP values (measured as height in milli-absorbance units, mAU) were normalized using ATP as the internal standard. The data represent the mean values from three biological replicates, with SEM calculated accordingly.

^b^ The extraction efficiency was determined to be 65% using standards.

### Distinct mutation patterns and hotspots in y252a and q255a mutants

**Figure 5.**
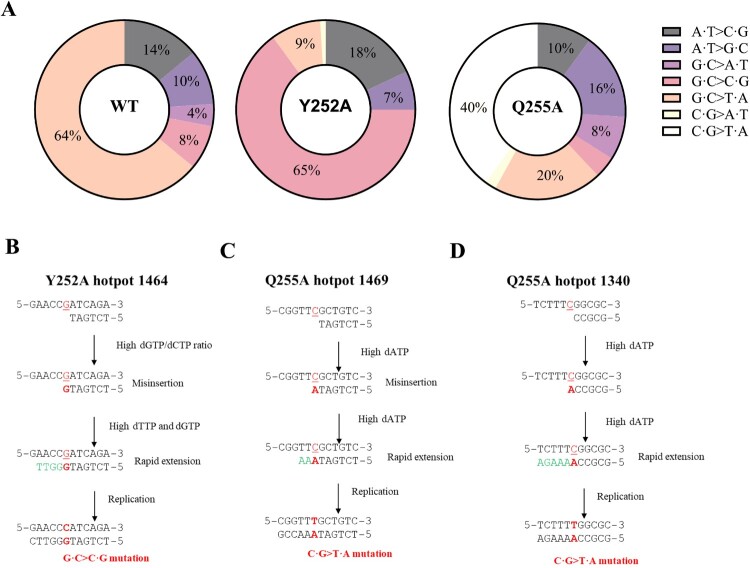
Distinct Mutation Patterns and Hotspots in *Y252A* and *Q255A* mutants. (A) Mutation spectra, expressed as a percentage of total mutations, in the cluster I and II regions of the *rpoB* gene. (B-D) Examples of predicted mutational mechanisms associated with observed mutation hotspots in *Y252A* (B) and *Q255A* (C, D). Red characters represent the mutational event and green bases represent bases where the dNTP is at an excessively high concentration.

Most RIF-resistance mutations occur in the *rpoB* resistance-determining regions (clusters I and II) [40]. We sequenced clusters I and II from 50 RIF-resistant colonies of each strain to identify mutation sites and types. Mutation incidence was calculated by multiplying the event percentage by the total mutation rate. *Y252A* and *Q255A* mutants were more prone to base substitutions, with *Y252A* also exhibiting higher rates of multiple-base deletions and insertions (Table S4). The *Y252A* mutant had a 10-fold higher incidence of GC > CG base substitutions than *WT*, with frequent occurrences at *rpoB* position 1464 (35/50) (Figure 5A). In contrast, the *Q255A* strain showed a preference for GC > TA and CG > TA substitutions, predominantly at positions 1469 (5/50) and 1340 (15/50) (Figure 5A). These findings suggest that loop mutations *Y252A* and *Q255A* cause distinct dNTP imbalances, leading to specific patterns of spontaneous mutations, as discussed further below.

## Discussion

While it has long been known that *Mtb* has class Ib RNRs encoded by *nrdE* (*Rv3051c*) and *nrdF2* (*Rv3048c*) [41], studies have not yet explored how RNR’s dysfunction affects *Mtb* physiology and genomic stability, as both *nrdE* and *nrdF2* are essential for *Mtb* growth [27]. Using CRISPRi technology, we generated an induced NrdE knockdown strain and found that NrdE knockdown significantly inhibited *Msm* and *Mtb* growth (Figure 1). NrdE knockdown significantly impaired the growth of Mycobacterium (Figure 1C, D), and accordingly, slow-growing or no-growing bacteria will enter a hypometabolic state [42], with down-regulation of expression of respiratory chain related-genes and reduced NAD^+^/NADH ratio (Figure 3A–C). These changes disrupted energy metabolism and redox homeostasis, leading to a significant increase in ROS levels (Figure 3E), while indirectly activating cellular compensatory mechanisms, including up-regulation of oxidative stress-related genes and enzymes to alleviate redox imbalance to minimize ROS-induced cellular damage [43,44] (Figure 3F–G).

In the hypometabolic state and with a low cellular energy level, *Mtb* does not carry out many biosynthetic activities, a major factor for its low susceptibility to antibacterials targeting protein, DNA, or cell wall biosynthesis [45]. Therefore, we hypothesize that the knockdown strain down-regulates own biosynthetic pathways into an energy-efficient state, such as reducing MA synthesis (Figure 2E–G), which indirectly increases bacterial permeability and alters antibiotic and environmental stress responses (Figure 2A–D, Figure S3). Given the critical role of NrdE in Mycobacterium growth and the fact that RNR dysfunction affects the expression of genes involved in important metabolic pathways, this suggests that NrdE is a promising target for the development of anti-tuberculosis drugs.

The nucleotide pool is highly susceptible to oxidative modification by ROS, which can lead to damaged genomic DNA through the incorporation of oxidized deoxynucleotides [36]. Endogenous ROS accumulation results in modified DNA bases, such as 8-oxo-7,8-dihydroguanine (8-oxo-G), 2-hydroxyadenine, and 5-hydroxycytosine [46–50], which can induce point mutations and even DNA breaks, destabilizing the genome [50]. Correspondingly, we observed an increased base mismatch rate in the NrdE knockdown strain (Figure 3M) and a twofold increase in rifampicin mutation frequency (Figure 3L), suggesting that NrdE knockdown leads to genomic instability and reduced DNA replication fidelity due to ROS accumulation. Surprisingly, the introduction of specific point mutations resulted in a much greater increase in mutation frequency, a 70-fold increase for *Msm-Y252A* and a 25-fold increase for *Msm-Q255A* (Figure 4F). Notably, genes related to DNA damage repair, including DSBR, NER, and BER, were significantly up-regulated after NrdE knockdown (Figure 3J–K); however, there was no activation of these mismatch repair systems observed in *Msm-Y252A* and *Msm-Q255A* (Figure 4G). We therefore hypothesize that activation of mismatch repair systems facilitates the repair of mutations in NrdE knockdown strains, perhaps explaining why NrdE knockdown resultes in a lower frequency of rifampicin mutations than that of the NrdE S-site single-point mutate strain. This also suggests that NrdE dysfunction leads to genomic instability and reduced DNA replication fidelity, and could even overwhelm active mismatch repair systems.

The dNTP pool balance is regulated by RNR’s S-site, with key amino acid residues in the loop domain linking the S-site and C-site [6]. Mutations in this loop domain can disrupt allosteric regulation, leading to dNTP imbalance [17]. We found that both NrdE *Y252A* and *Q255A* mutations in the S-site led to a significant increase in the spontaneous RIF mutation frequency (Figure 4F, Table S4) but with different effects on dNTP concentrations (Table 2). In the *Msm*-*Q255A* mutant, all dNTP levels were elevated except dCTP, which unchanged. The Q288 residue in eukaryotic RNRs, homologous to Q255 in *Msm*, directly interacts with all substrates except GDP [51]. We propose that the Q255 mutation, similar to Q288, reduces the binding of all substrates except GDP, raising dGTP levels, which in turn elevates dATP. For Y252A, weakened dGTP binding likely enhances CDP and UDP reduction while impairing ADP reduction [18], increasing dCTP and dTTP levels with minimal impact on dATP in *Msm*-Y252A. Whether and how the mutations affect the allosteric regulation of the Mycobacterial RNR remains to be investigated. These dNTP imbalances led to distinct mutation patterns and hotspots. The mutation hotspot in *Y252A*, particularly GC > CG substitutions at *rpoB* position 1464, likely results from misinsertion due to a high dGTP/dCTP ratio, followed by rapid extension facilitated by high dTTP and dGTP levels (Figure 5B) [52,53]. In *Q255A*, CG > TA substitutions at positions 1469 and 1340 may stem from dATP misinsertion and extension driven by elevated dATP and dGTP levels (Figure 5C–D).

Acquired genetic mutations are the primary cause of drug-resistant *Mtb* emergence [54]. Although *Msm-Y252A* and *Msm-Q255A* strains did not show increased resistance to tested antibiotics (Table S5), their elevated mutation rates remain a critical factor for acquired drug resistance in *Mtb* [2]. Interestingly, despite only a modest dNTP imbalance (1.3–9.0 – fold), RIF-resistant mutation rates increased 70-fold and 25-fold in *Msm-Y252A* and *Msm-Q255A*, respectively, indicating that dNTP pool homeostasis plays a key role in maintaining genomic stability and mutability in Mycobacterium, and that even slight dNTP imbalances can drive mutagenesis. These have also been found in *E.coli* and yeast [26,39,55,56]*,* The G295S and A301 V mutator alleles of *E. coli* RNR suffer from a 1,000-fold or more increase in forward mutations in the *rpoB* gene, while having modest dNTP pool changes that include approximately twofold changes in the concentration of dGTP (increased) and dATP (reduced) [17]. Another study demonstrates that overexpression of RNR in *E. coli* resultes in a threefold increase in the dATP, dCTP, and dTTP pools, while the dGTP pool remaines unaffected. This imbalance ultimately leads to a 30-fold rise in spontaneous mutation rates to rifampicin resistance [56]. Certainly, the determination of spontaneous mutation rate can also be affected by different methods [5,57,58]. Here, the fluctuation assay relies on the selection of rifampicin-resistant mutants that exhibit *rpoB* mutation, since the RIF^R^ phenotype is mainly (over 95%) existed in the cluster I and II regions of *rpoB* [59], the mutations detected using this method may not yet be representative of the whole-genome. Genome-wide unbiased mutations may be better reflected by mutations accumulation in combination with whole-genome sequencing [60]. In particular, we failed to obtain genomic mutant strains with NrdE S-site mutation found in clinical resistant strains. The effect of these naturally selected mutations on dNTP homeostasis and their roles in the evolution of drug resistance in *Mtb* warrant further investigation.

Overall, our findings demonstrate that NrdE is crucial for regulating key metabolic processes and maintaining genome stability in *Mycobacterium*. It suggests that RNR dysfunction indirectly fosters drug resistance by creating a mutagenic environment under selective pressure, positioning NrdE as a key target for therapeutic strategies and a biomarker for early detection of drug-resistant tuberculosis.

## Author contributions

Xuelian Zhang conceived the study and designed experiments. Xiao Zhang performed the experiments and analysed the data. Yuchang Di contributed to methodology and performed TLC experiments. Yu Zhang assisted in the analysis of *Mtb* resistant strains. Youwei Hu, Mingzhe Chi, Jian Kang, Yuqing Zheng, Hengyu Wang and Yu Wang assisted in bacterial phenotype experiments. Xuelian Zhang and Xiao Zhang wrote the manuscript, and all authors commented on the manuscript, data, and conclusion.

## Supplementary Material

Supplementary_Figures_revised_version-clean.doc

Supplementary Tables_revised version.docx
