# Supplementary material for: Hypermutability of Mycolicibacterium smegmatis due to ribonucleotide reductase-mediated oxidative homeostasis and imbalanced dNTP pools
Source: Emerg Microbes Infect. 2025 Mar 18;14(1):2480698. doi: 10.1080/22221751.2025.2480698 (PMC11948356; doi:10.1080/22221751.2025.2480698)
Supplement: Supplementary_Figures_revised_version-clean.doc [file TEMI_A_2480698_SM9567.doc]

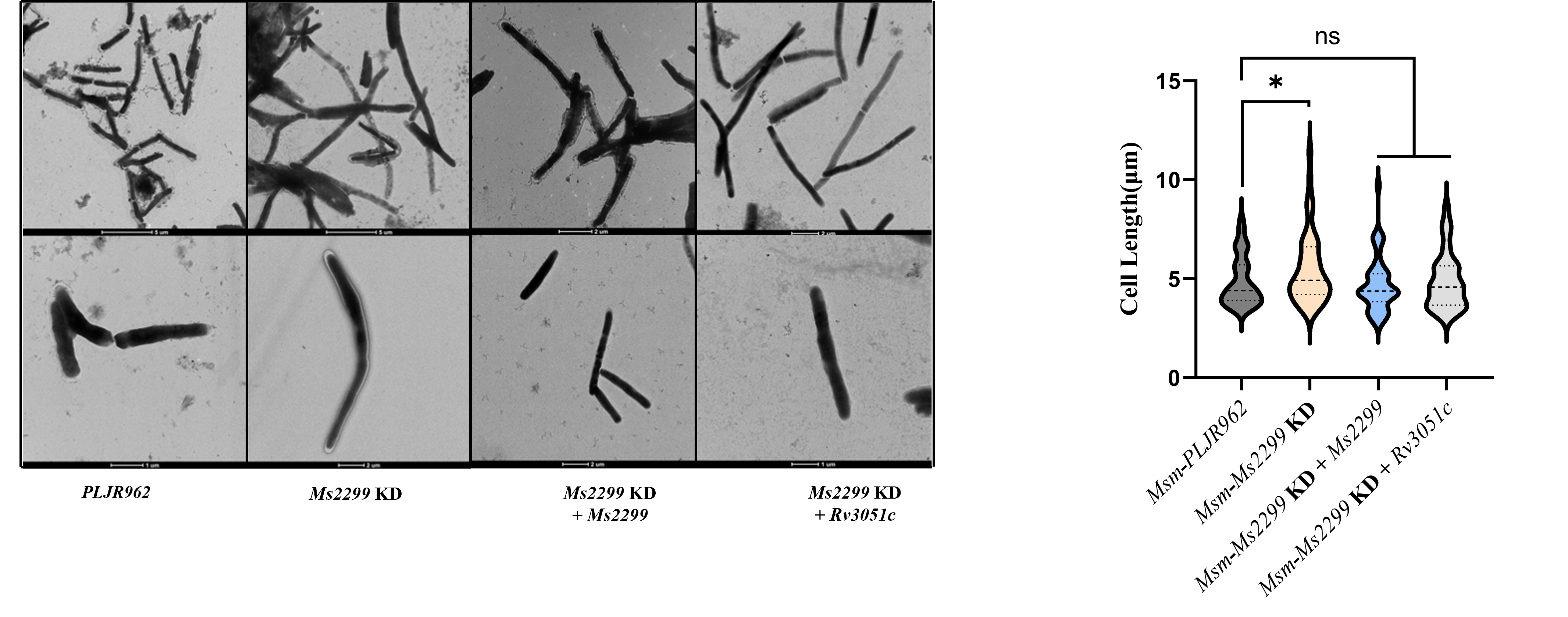


**Figure S1.** Morphological observations and lengths of *Msm.* The data reported represent the means (n = 50) ±SD (standard deviation). ns, not significant; *, *p* < 0.05.


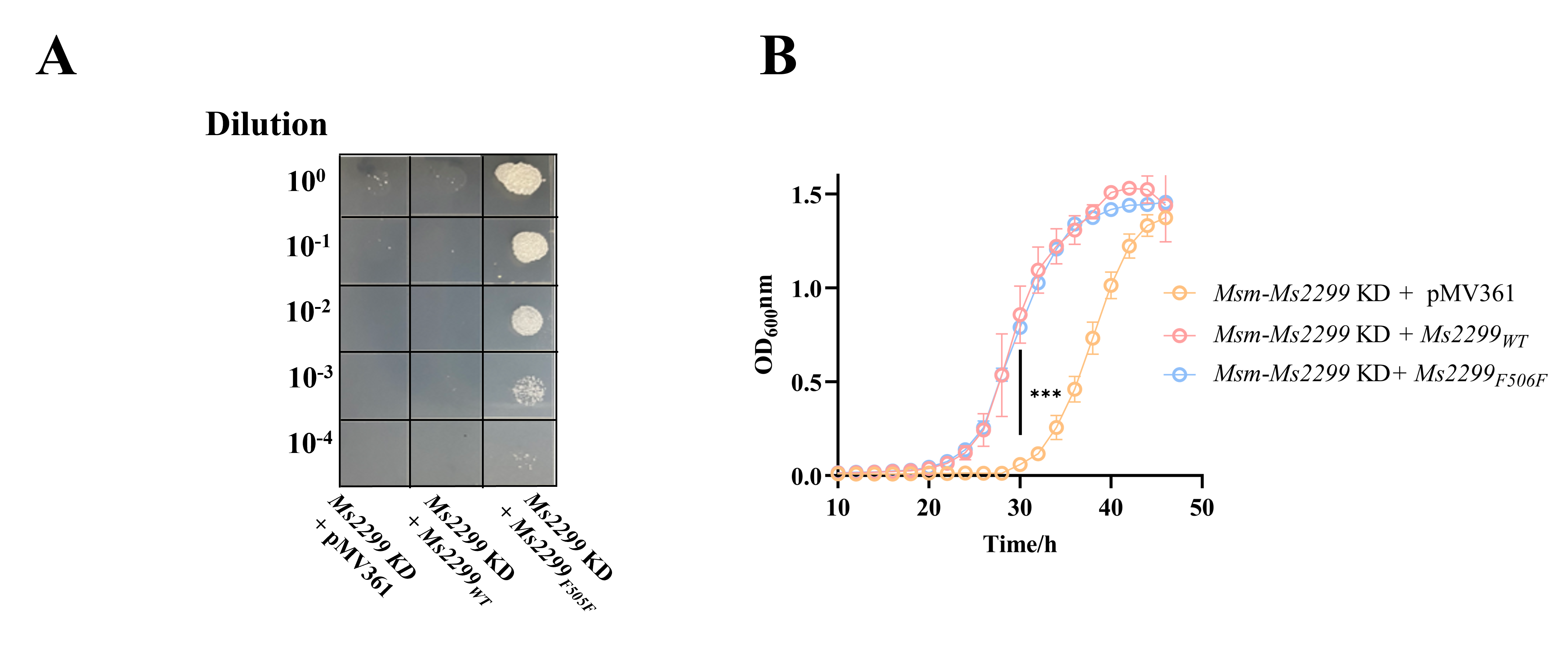


**Figure S2.** Growth inhibition in *Msm* due to *Ms2299* knockdown could be restored by introducing *Ms2299_F505F_*, while the introduction of *Ms2299_WT_* did not. (A) Ten-fold serial dilutions of *Msm* complementation strains were spotted on Middlebrook 7H10 with ATC. (B) Growth curves of *Msm* strains in 7H9-OADC medium with ATC. Data represent the mean (*n* = 3) ± SD. ***, *p* < 0.001.


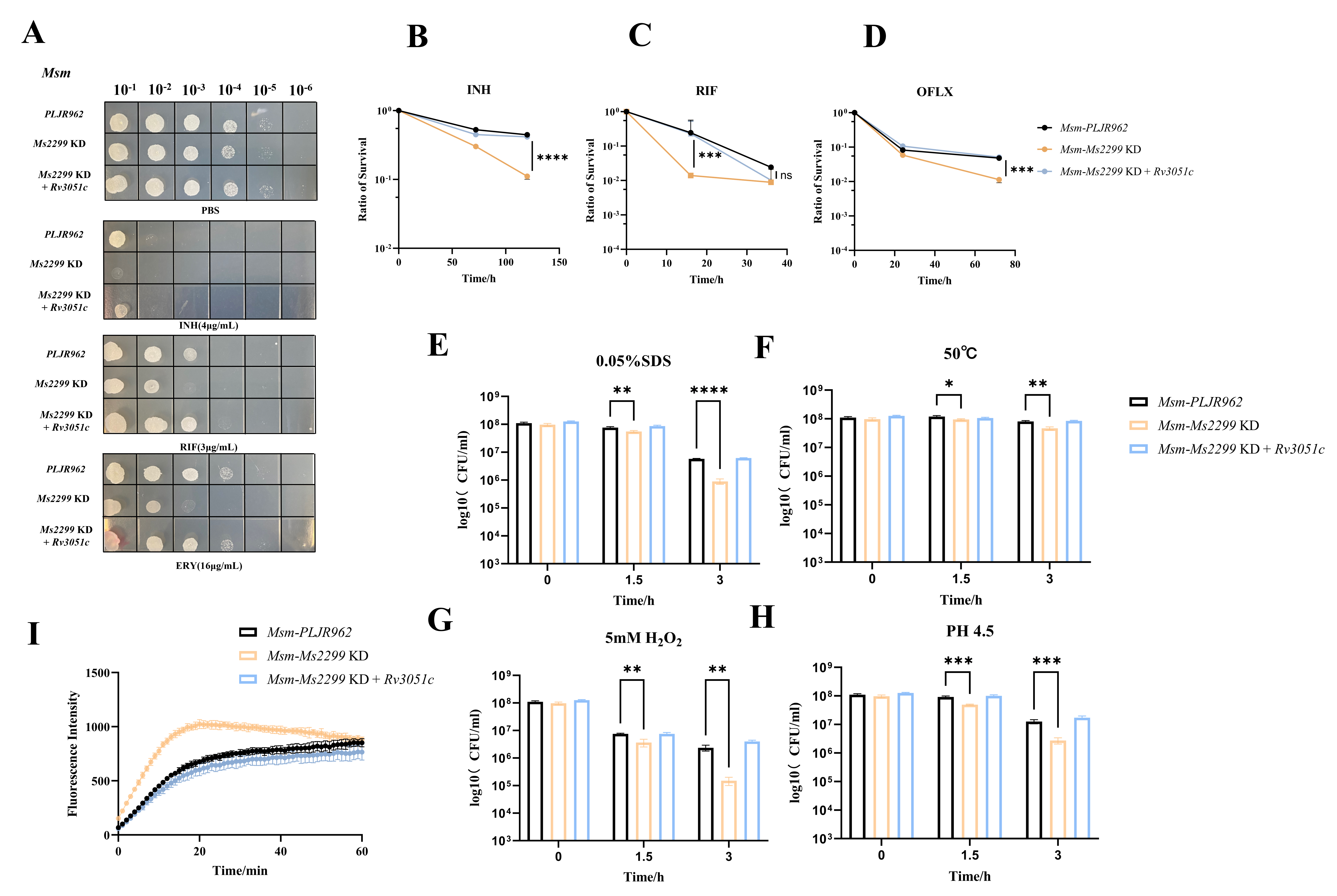


**Figure S3.** Introducing *Rv3051c_F509F_* rescued the growth defects, drug susceptibility, and environmental stress-related phenotypes of *Msm-Ms2299* KD. (A) Ten-fold serial dilutions of *Msm* strains were spotted on 7H10 medium containing PBS, INH (4 μg/ml), RIF (3 μg/ml) and ERY (16 μg/ml). (B-D) Survival of *Msm* strains exposed to lethal concentrations of antibiotics (10× MIC), including INH (B), RIF (C) and OFLX (D). (E-H) Survival of *Msm* strains exposed to different stress conditions, including 0.05% SDS (E), heat treatment (F), 5 mM H_2_O_2_ (G) and low pHs of 4.5 (H). (I) Log-phase cultures of *Msm* strains (complement with *Ms2299*) incubated in PBS with 0.04% glucose and 2 μg/mL EB; fluorescence was measured every minute for one hour. Data represent the mean (n = 3) ± SD. ns, not significant; *, *p* < 0.05, **, *p* < 0.01, ***, *p* < 0.001, ****, *p* < 0.0001.


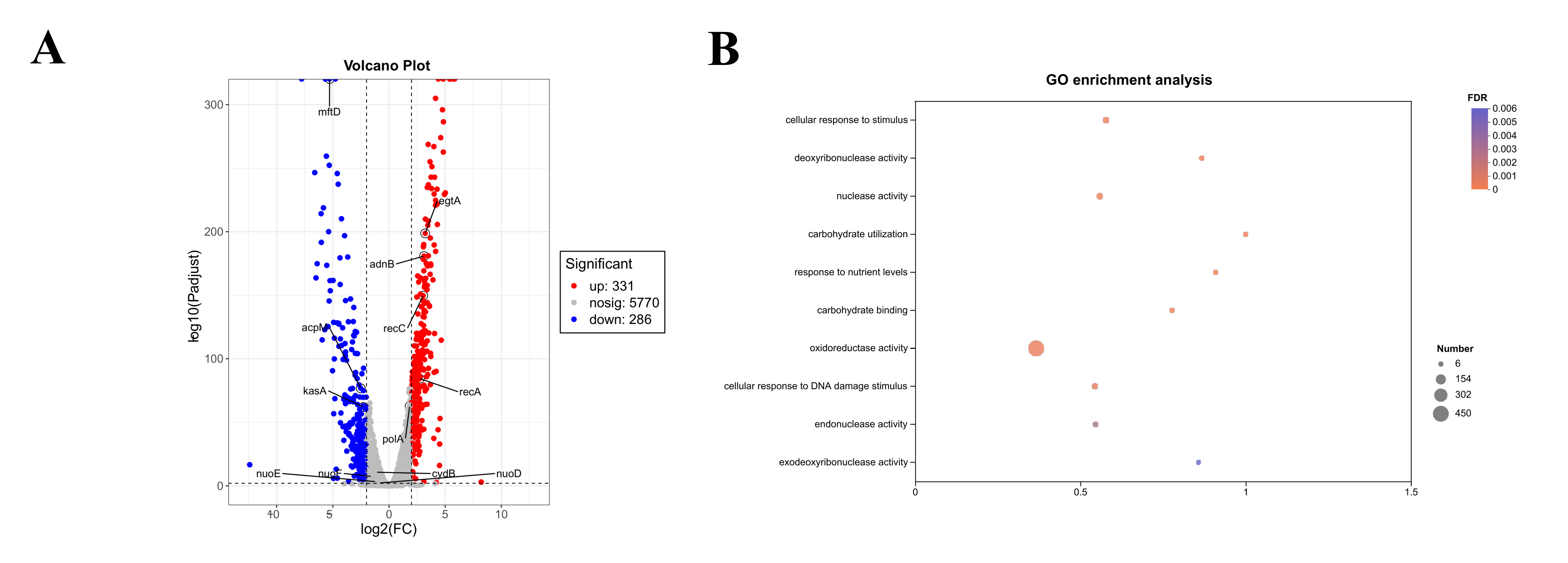


**Figure S4.** (A) Volcano plot showing differentially expressed genes between *Msm*-*PLJR962* and *Msm*-*Ms2299* KD (*p* < 0.01 and |log_2_ FC| ≥ 2). Differential expression between groups was analyzed by Majorbio Cloud Platform. (B) GO enrichment analysis shows the enrichment partial results of the top20. The horizontal Rich factor indicates the enrichment degree, the size of the points indicates the number of genes/transcripts in this GO Term, and the color of the points corresponds to different FDR (Pvaule_corrected) ranges.


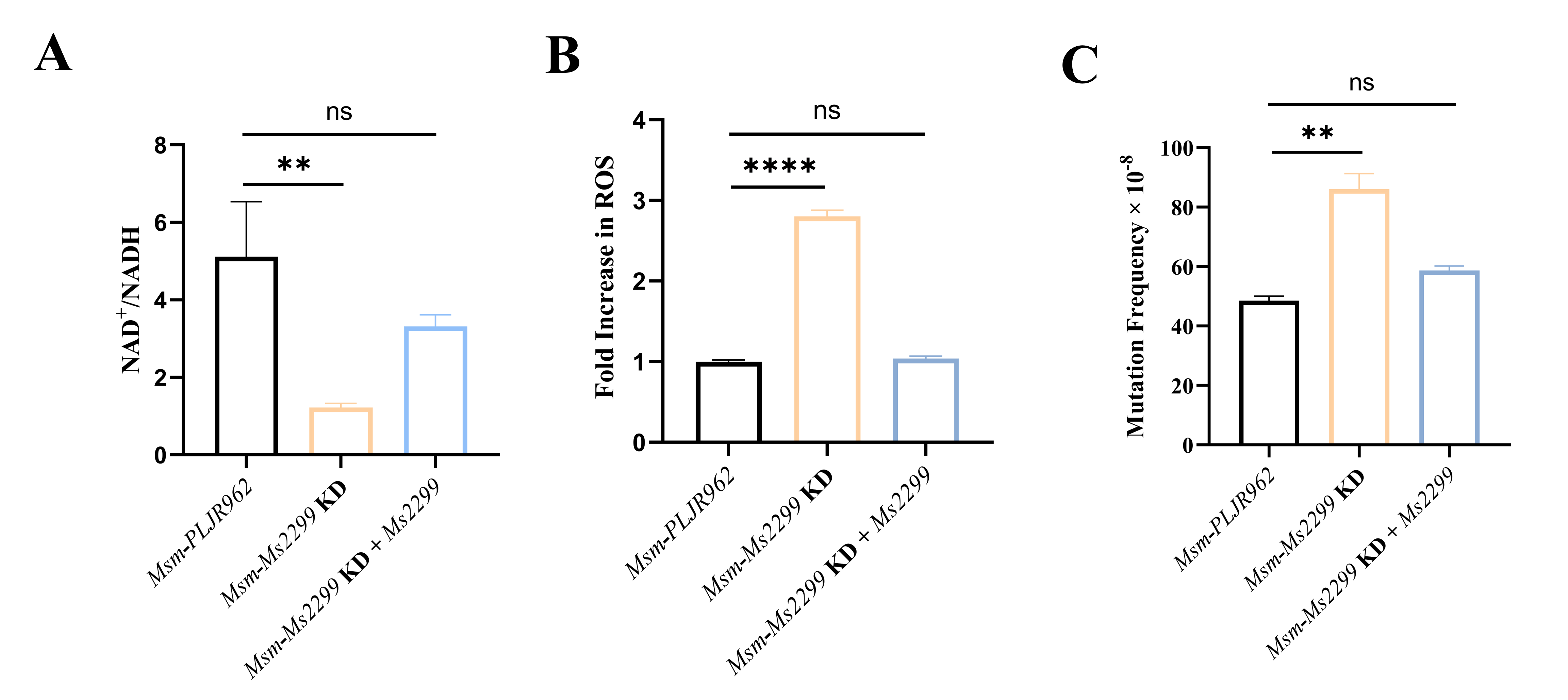


**Figure S5.** (A) Comparison of NAD^+^/NADH radios and (B) ROS level in *Msm*-*PLJR962*, *Msm*-*Ms2299* KD*,* and *Msm*-*Ms2299* KD + *Ms2299.* (C) Frequency of RIF^R^ mutations in *Msm*-*PLJR962*, *Msm*-*Ms2299* KD*,* and *Msm*-*Ms2299* KD + *Ms2299*. Data represent the mean (n = 3) ± SD. ns, not significant, **, *p* < 0.01, ****, *p* < 0.0001.
