# Supplementary material for: Hypermutability of Mycolicibacterium smegmatis due to ribonucleotide reductase-mediated oxidative homeostasis and imbalanced dNTP pools
Source: Emerg Microbes Infect. 2025 Mar 18;14(1):2480698. doi: 10.1080/22221751.2025.2480698 (PMC11948356; doi:10.1080/22221751.2025.2480698)
Supplement: Supplementary Tables_revised version.docx [file TEMI_A_2480698_SM9566.docx]

**Table S1 *nrdE* (*Rv3051c*) mutations identified in 1393 drug-resistant strains of *Mtb.***

| **Locus** | **Codon** | **Nucleotide change** | **Amino change** | **No. of strains** | **Percentage** |
| --- | --- | --- | --- | --- | --- |
| 3412611 | 551 | CAG-CGG | Gln/Q-Arg/R | 1181 | 84.8 |
| 3413785 | 160 | GTC-ATC | Val/V-Ile/I | 184 | 13.2 |
| 3413574 | 230 | GCG-GGG | Ala/A-Gly/G | 9 | 0.64 |
| 3412218 | 682 | GCC-GTC | Ala/A-Val/V | 8 | 0.57 |
| 3412966 | 433 | ATC-GTC | Ile/I-Val/V | 3 | 0.22 |
| 3412405 | 620 | GTC-ATC | Val/V-Ile/I | 2 | 0.22 |
| 3412698 | 522 | CGC-CAC | Arg/R-His/H | 2 | 0.22 |
| 3412711 | 518 | CGG-TGG | Arg/R-Trp/W | 2 | 0.22 |
| 3412759 | 502 | ATC-GTC | Ile/I-Val/V | 1 | 0.11 |
| 3412882 | 461 | CAT-TAT | His/H-Tyr/Y | 1 | 0.11 |

**Table S2 *nrdE* (*Rv3051c*) mutations identified in 978 drug-sensitive strains of *Mtb.***

| **Locus** | **Codon** | **Nucleotide change** | **Amino change** | **No. of strains** | **Percentage** |
| --- | --- | --- | --- | --- | --- |
| 3412611 | 551 | CAG-CGG | Gln/Q-Arg/R | 69 | 7.06 |
| 3413785 | 160 | GTC-ATC | Val/V-Ile/I | 160 | 16.36 |
| 3412405 | 620 | GTC-ATC | Val/V-Ile/I | 10 | 1.02 |
| 3413100 | 356 | CCA-CTA | Pro/P-Leu/L | 3 | 0.31 |

**Table S3 Primers**

| Primer | Sequence | Purpose |
| --- | --- | --- |
| sg3051c-F | AAACCAAGAAGCAGCGCGGGGAGC | sgRNA for *Mtb* *Rv3051c* knock down |
| sg3051c-R | GGGAGCTCCCCGCGCTGCTTCTTG |  |
| q3051c-F | TACCTGTTCTCGCCCTACGA | Δ*Rv3051c* qPCR check |
| q3051c-R | GAAGAACTCCCGTGCCTTGA |  |
| sg2299-F | GGGAAGCGCGTGGAACAGCACCGT | sgRNA for *Msm* *Ms2299* knock down |
| sg2299-R | AAACACGGTGCTGTTCCACGCGCT |  |
| q2299-F | GTTCAACGACGACCTGTCCT | Δ*Ms2299* qPCR check |
| q2299-R | TCTGCACCGACTCCTTCAAC |  |
| qMs-sigA-F | CGTCCGGCGACTTCGTGT | *Msm* qPCR |
| qMs-sigA-R | TGGCCAGCTCCACCTCTTCT |  |
| qMtb-sigA-F | CTCGACGCTGAACCAGAC | *Mtb* sigA qPCR |
| qMtb-sigA-R | GAGGTCTTCGTGGTCTTCGT |  |
| cr-loop-F | TCCTACGCCAATCAGCTGGGTGCCC | *Msm* crRNA for loop domain mutations |
| cr-loop-R | GGGCACCCAGCTGATTGGCGTAGGA |  |
| ssDNA-252 | CATGAAGCTGCTGGAGGACTCGTTCTCCGCAGCCAATCAGCTGGGTGCCCGTCAGGGCG | ssDNA for *Y252A* mutation |
| ssDNA-255 | ACCGCGCCCGCGCCCTGACGGGCACCCAGCGCATTGGCGTAGGAGAACGAGTCCTCCAG | ssDNA for *Q255A* mutation |
| loop-F | TCCATCAACTCGGCGCTGCA | *Y252A* and *Q255A* check |
| loop-R | TAGACGCGCTCGACGTCGTA |  |
| rpoB-F | GCTGATCCAGAACCAGATCC | *rpoB* mutation spectrum |
| rpoB-R | GATGACACCGGTCTTGTCG |  |
| 361-2299-F | GCGGATCCAGCTGCAGAATTCGTGCCACCCACCGTCACA | *Ms2299* complement |
| 361-2299-R | TACGTCGACATCGATAAGCTTTCACAGCATGCAGCTGACGC |  |
| 361-3051c-F | CAGCTGCAGAATTCGAAGCTTGTGCCACCAACCGTCATTGC | *Rv3051c* complement |
| 361-3051c-R | GTTAACTACGTCGACATCGATTTAGTGGTGATGGTGATGATGCAGCATGCAGGACACGCAA |  |
| mut-2299cr-F | ACGAACATCTACTTTTACACGGTGCTGTTCCAC | PAM on pMV361-drived constructs was synonymous mutated to prevent sgRNA binding |
| mut2299cr-R | GAAGTCGATGCCCTCTTCGGAACCGT |  |
| mut3051cr-F | ACCAACATCTACTTTTATACGGTGCTGTATCACGC |  |
| mut3051cr-R | GAAGTCGATGCCTTCGTCGGATCC |  |
| kasA-F | GGAATCGATCCTCACGGTCC | *Msm* qPCR |
| kasA-R | CGAATGCCAGAGCCACATTG |  |
| acpM-F | GACGACCTGGACATCGACTC | *Msm* qPCR |
| acpM-R | TGGATGTAGGCAACCACGTC |  |
| mftD-F | CGGCTTCCGAAATCCGTCTA | *Msm* qPCR |
| mftD-R | GAGATGATGACCGGCAACGA |  |
| qcrA-F | AAGGGCGAGACCATCTACCT | *Msm* qPCR |
| qcrA-R | GATCTCGGTCAGCTTGTGCT |  |
| mbtD-F | CATCGACGCAGACGAGATCA | *Msm* qPCR |
| mbtD-R | GTGCCGAGAAAAAGTGCGAG |  |
| cydB-F | CAAGCAGATCCACCTGTCGT | *Msm* qPCR |
| cydB-R | GAGGTCTTCAGCGACACGAA |  |
| trxA-F | CGTGCATGGCAAAGTCAACA | *Msm* qPCR |
| trxA-R | AAGGTGTATGCCCGGAGTTG |  |
| trxB-F | GACGTTCCTCACACGCTTTG | *Msm* qPCR |
| trxB-R | CGATCTGGGTGATCTCGGTG |  |
| msrA-F | ATCCGACGTACCGCAAT | *Msm* qPCR |
| msrA-R | ACGTCGTTGCCCTGA |  |
| egtA-F | CGGTGATGCTGGTGAACTCT | *Msm* qPCR |
| egtA-R | GATAGCGGATCTCCAGCCAG |  |
| sodA-F | CCAGCAAGCAAATGTCCCAC | *Msm* qPCR |
| sodA-R | CCAGTTGACGACGTTCCAGA |  |
| aphc-F | GGTGACCAGTTTCCGGAGTA | *Msm* qPCR |
| aphc-R | TCGAAGTCCTCGTTCAGCTT |  |
| aphD-F | TCGCACCGAGATCAACTACG | *Msm* qPCR |
| aphD-R | TTGCATGGAGGAACTCGGAC |  |
| polA-F | GCCGAAGACCAAGAAGACCA | *Msm* qPCR |
| polA-R | TGGTTGAACGTCGTGTGGAT |  |
| adnB-F | CAGGACGCGTTCGTCAAATC | *Msm* qPCR |
| adnB-R | CCGGTCTTCCAGTCGAGAAC |  |
| ligA-F | AGGACTCTCGATCGTGGTGA | *Msm* qPCR |
| ligA-R | CCACGTAGGCGGTCTTCTTC |  |
| nei2-F | GAGGGACATGTTGTGGCTCA | *Msm* qPCR |
| nei2-R | ATCGGTCTGGATCAACGTGC |  |
| nth-F | GGCGAAACCAAAGAAGTCGG | *Msm* qPCR |
| nth-R | AAGTCCAGCTCGCAGTACAC |  |
| recA-F | GTCATCGAGATCTACGGCCC | *Msm* qPCR |
| recA-R | GTGTCCACACCCAGCTTCTT |  |
| recC-F | CAAACGCAACGTGATCCCTG | *Msm* qPCR |
| recC-R | GCAACGGATCGCTGAAGAAC |  |
| urvA-F | TCGAGAAGCTCAACAGCCAG | *Msm* qPCR |
| urvA-R | GTCGTGCTTCTCCTGCTTCT |  |
| urvB-F | AGGACAGCTCGATCAACGAC | *Msm* qPCR |
| urvB-R | AACGGTCCAGATACGACTGC |  |

**Table S4. Mutation rates of specific mutation events in spontaneous RIF^R^ mutation**

|  | Mutation rate ×10^8^ | | |
| --- | --- | --- | --- |
|  | *WT* | *Y252A* | *Q255A* |
| Totals | 1.76 | 122.5 | 44.66 |
| Base substitution | 1.76 | 171.5 | 44.66 |
| Transition | 0.352 | 14.7 | 13.4 |
| Transversion | 1.408 | 120.0 | 31.26 |
| Indels | 0 | 8.75 | 2.67 |
| Deletions | 0 | 10.5 | 0.89 |

| Strain | MIC(μg/mL) | | | | | | | |
| --- | --- | --- | --- | --- | --- | --- | --- | --- |
|  | INH | RIF | OFLX | CFZ | STR | ERY | FA |  |
| *Msm*-*WT* | 12.5 | 9.3 | 0.312 | 5 | 0.625 | 40 | 128 |  |
| *Msm*-*Y252A* | 50 | 9.3 | 0.312 | 5 | 0.625 | 40 | 128 |  |
| *Msm*-*Q255A* | 25 | 18.7 | 0.312 | 5 | 0.625 | 40 | 128 |  |

**Table S5. MIC of various antibiotics for *Msm*-*WT*, *Msm*-*Y252A*, *Msm*-*Q255A*.** INH, isoniazid; RIF, rifampicin; OFLX, ofloxacin; CFZ, clofazimine; STR, streptomycin; ERY, erythromycin; FA, fusidic acid.
